# A Transformed Bacterium Expressing Double-Stranded RNA Specific to Integrin β1 Enhances Bt Toxin Efficacy against a Polyphagous Insect Pest, *Spodoptera exigua*

**DOI:** 10.1371/journal.pone.0132631

**Published:** 2015-07-14

**Authors:** Eunseong Kim, Youngjin Park, Yonggyun Kim

**Affiliations:** Department of Bioresource Sciences, Andong National University, Andong, 760–749, Republic of Korea; Institute of Plant Physiology and Ecology, CHINA

## Abstract

**Background:**

Oral toxicity of double-stranded RNA (dsRNA) specific to integrin β1 subunit (*SeINT*) was known in a polyphagous insect pest, *Spodoptera exigua*. For an application of the dsRNA to control the insect pest, this study prepared a transformed *Escherichia coli* expressing dsRNA specific to *SeINT*.

**Principal Findings:**

The dsRNA expression was driven by T7 RNA polymerase overexpressed by an inducer in the transformed *E*. *coli*. The produced dsRNA amount was proportional to the number of the cultured bacteria. The transformed bacteria gave a significant oral toxicity to *S*. *exigua* larvae with a significant reduction of the *SeINT* expression. The resulting insect mortality increased with the fed number of the bacteria. Pretreatment with an ultra-sonication to disrupt bacterial cell wall/membrane significantly increased the insecticidal activity of the transformed bacteria. The larvae treated with the transformed bacteria suffered tissue damage in the midgut epithelium, which exhibited a marked loss of cell-cell contacts and underwent a remarkable cell death. Moreover, these treated larvae became significantly susceptible to a Cry toxin derived from *Bacillus thuringiensis* (Bt).

**Conclusions:**

This study provides a novel and highly efficient application technique to use dsRNA specific to an integrin gene by mixing with a biopesticide, Bt.

## Introduction

Application of double-stranded RNA (dsRNA) has been focused on controlling specific target insect pests in agriculture [1]. RNA interference (RNAi) induced by exogenous dsRNA leads to loss of a specific function in insect physiological processes and results in a significant threat to survival. Various target genes have been screened to develop an efficient dsRNA insecticide. A V-ATPase gene, which is expressed in the midgut of the corn rootworm, *Diabrotica virgifera virgifera*, was targeted by its specific dsRNA and gave a significant mortality to the pest [2]. Furthermore, to be specific to insects and other arthropods, a chitin synthase was targeted to develop dsRNA insecticide [3,4]. Targeting ecdysone receptor by dsRNA is another example for the specific control insect pests to avoid any detrimental impact on mammals [5].

Delivery techniques of the efficient dsRNAs to target insects especially into cytoplasm have been investigated. The environmental RNAi is highly efficient by injecting dsRNA directly to the hemocoel of target insects [6]. For its practical purpose, feeding application of dsRNA has been demonstrated in several insects with little information of the route of dsRNA into the target cytoplasm [7–9]. To give a stability of dsRNA in environmental condition, nanoparticle formulation of dsRNA was applied to aquatic condition and proved for its application to control mosquitoes [4]. On the other hand, a formulation of dsRNA in bacteria can give dual benefits in terms of dsRNA synthesis and stability in a kind of bioformulation. A recombinant bacteria expressing dsRNA gave significant RNAi efficacy and resulted in target insect mortality by feeding the bacteria in the Colorado potato beetle, *Leptinotarsa decemlineata* [10]. Furthermore, transgenic plants expressing dsRNA in a hairpin form have been constructed and are effective to give significant RNAi efficacy [11–13].

The beet armyworm, *Spodoptera exigua*, is a serious insect pest on vegetable crops with its wide host range [14,15]. Its development of resistance against various chemical and microbial insecticides needs an alternative control technique [16–18]. Several genes of *S*. *exigua* are modulated in their expressions by their specific dsRNA treatments and exhibit significant RNAi efficacies [19–24]. Moreover, oral feeding of dsRNA was effective to induce significant RNAi in *S*. *exigua* [23,25].

Integrin is a heterodimer transmembrane protein and plays a crucial role in cell-cell and cell-extracellular matrix (ECM) interactions [26]. A β subunit of integrin (SeINT) has been identified in *S*. *exigua* and is associated with cellular immune responses and larval development [27]. Injection or oral treatment of *in vitro* prepared dsRNA specific to *SeINT* gave significant mortality to *S*. *exigua* larvae [27].

This study aimed to develop a dsRNA insecticide against *S*. *exigua* by constructing a transformed bacterium. To this end, *SeINT* gene expression was targeted by its specific dsRNA expressed in *Escherichia coli* HT115 lacking in RNase ΙΙΙ. To enhance the insecticidal activity, the dsRNA-producing bacteria were mixed with *E*. *coli* expressing a Cry toxin of *Bacillus thuringiensis* (Bt). This study introduces a novel application of dsRNA potentiating Bt toxicity.

## Results

### Transformed bacteria expressing dsRNA specific to integrin β1 subunit of *S*. *exigua*

**Fig 1:**
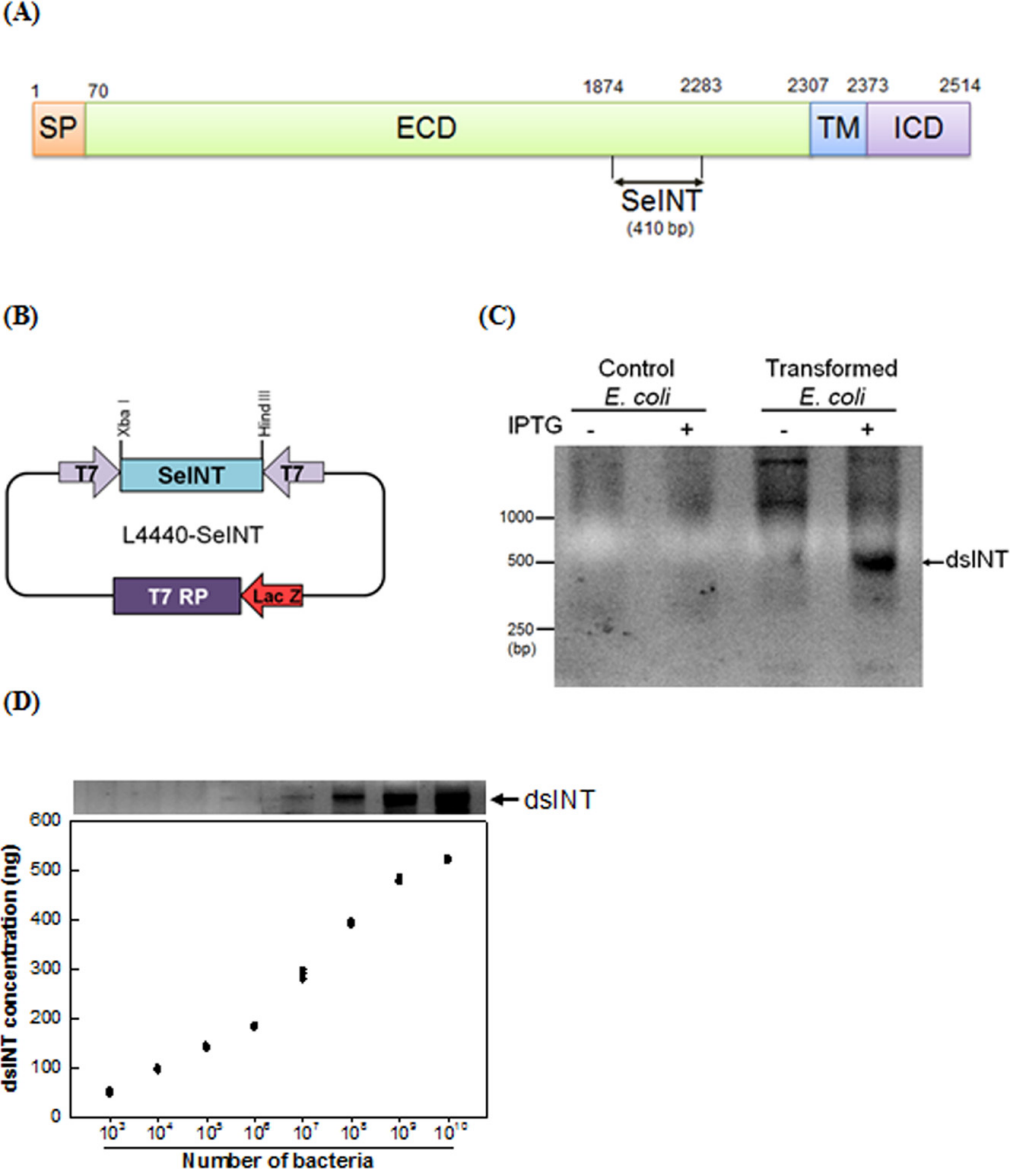
Construction of a recombinant *E*. *coli* expressing dsRNA (‘dsINT’) specific to an integrin β1 subunit fragment (‘SeINT’). (A) A diagram showing a fragment (410 bp) used for generating dsINT: signal peptide (‘SP’), extracellular domain (‘ECD’), transmembrane (‘TM’), and intracellular domain (‘ICD’). (B) Cloning SeINT into an expression vector of L4440. Lactose promotor (‘Lac Z’) directs the expression of T7 RNA polymerase (‘T7 RP’). SeINT is transcribed by both directions according to T7 RNA polymerase promoters (‘T7’). (C) Production of dsINT with an inducer, IPTG. Transformed *E*. *coli* with L4440-SeINT expressed dsINT at the presence of IPTG. (D) Quantification of dsINT according to the transformed bacterial cell numbers. The extracted dsINT was quantified by band intensity based on a standard curved using the known amounts of purified dsINT prepared by *in vitro* dsRNA construction method. Each measurement was replicated three times.

Test bacteria used in this study were prepared with a recombinant vector containing a fragment of *S*. *exigua* β1 integrin gene (‘SeINT’, Fig 1). A PCR product (Fig 1A) of an integrin fragment at its extracellular domain was inserted between two T7 RNA polymerase promoters (Fig 1B). The recombinant vector was used to transform *E*. *coli* HT115 lacking RNase ΙΙΙ. The transformed *E*. *coli* was induced to overexpress dsRNA under LacZ promoter by adding an isopropyl β-D-thiogalactoside (IPTG) inducer (Fig 1C). The induced bacteria expressed dsRNA specific to the integrin (‘dsINT’) and the amount of produced dsRNA was linearly related with the bacterial cell number in log scale (dsRNA in ng = 31 x Log (Bacterial cell number)– 199, R^2^ = 0.9756) (Fig 1D).

### Insecticidal activity of dsINT-expressing *E*. *coli* against *S*. *exigua*

**Fig 2:**
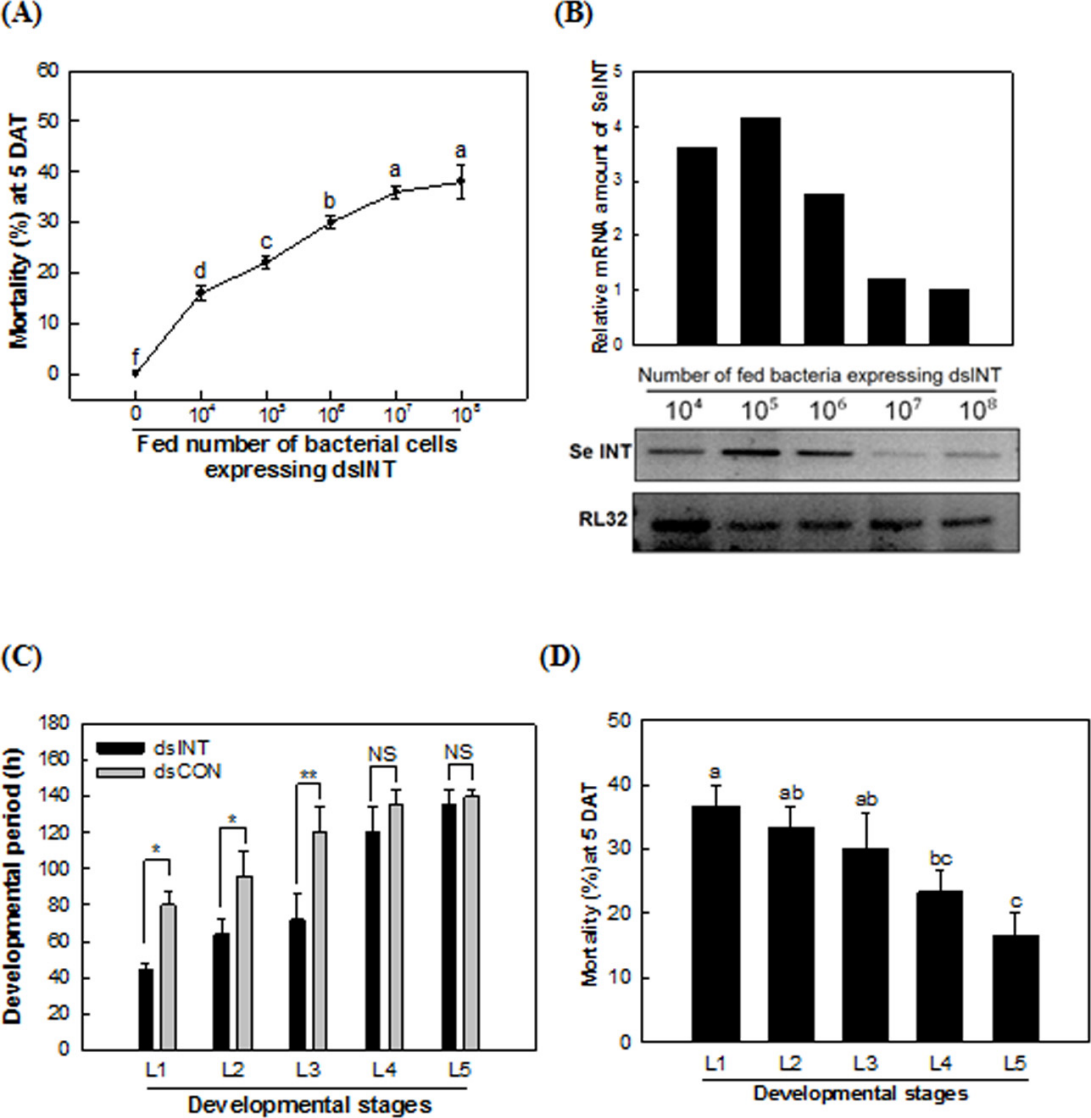
Insecticidal activity of a transformed *E*. *coli* expressing dsRNA (‘dsINT’) specific to an integrin β subunit fragment (‘SeINT’). (A) Oral toxicity of the recombinant bacteria (10^7^ cells per larva) to young larvae. Each dose test used 10 larvae with three replications. Mortality was measured after 5 days of treatment (‘DAT’). (B) Suppression of SeINT transcript levels measured by RT-PCR in the bacterial treatment. Expression of a ribosomal gene, RL32, was assessed to confirm the cDNA integrity. (C, D) Effects of the recombinant bacteria on developmental rate and toxicity. The recombinant bacteria (10^7^ cells per larva) were independently fed to each instar (‘L1-L5’) with 10 larvae per replication. Untreated instars were fed with fresh cabbage. Each treatment was replicated three times. The developmental period of each treated instar was measured. Mortality was measured at 5 DAT. Different letters above standard deviation bars indicated significant difference among means at Type Ι error = 0.05 (LSD test). ‘NS’ represents not significant, but ‘*’ and ‘**’ indicate significant differences among means at Type I error = 0.05 and 0.01, respectively.

The transformed bacteria were fed to larvae of *S*. *exigua* (Fig 2). The insecticidal activity of the transformed bacteria was observed and increased in a bacterial dose-dependent manner (Fig 2A). The transformed bacterial treatment suppressed the expression of *SeINT* at high bacterial doses (Fig 2B). Compared to control bacteria, dsINT-expressing bacteria delayed the larval development when they were fed at young instars (Fig 2C). Indeed, young instar larvae were more susceptible to the transformed bacterial treatment than old instar larvae (Fig 2D).

### Comparison of *in vitro* prepared dsRNA with the transformed bacteria expressing dsRNA in RNAi efficiency and insecticidal activity

**Fig 3:**
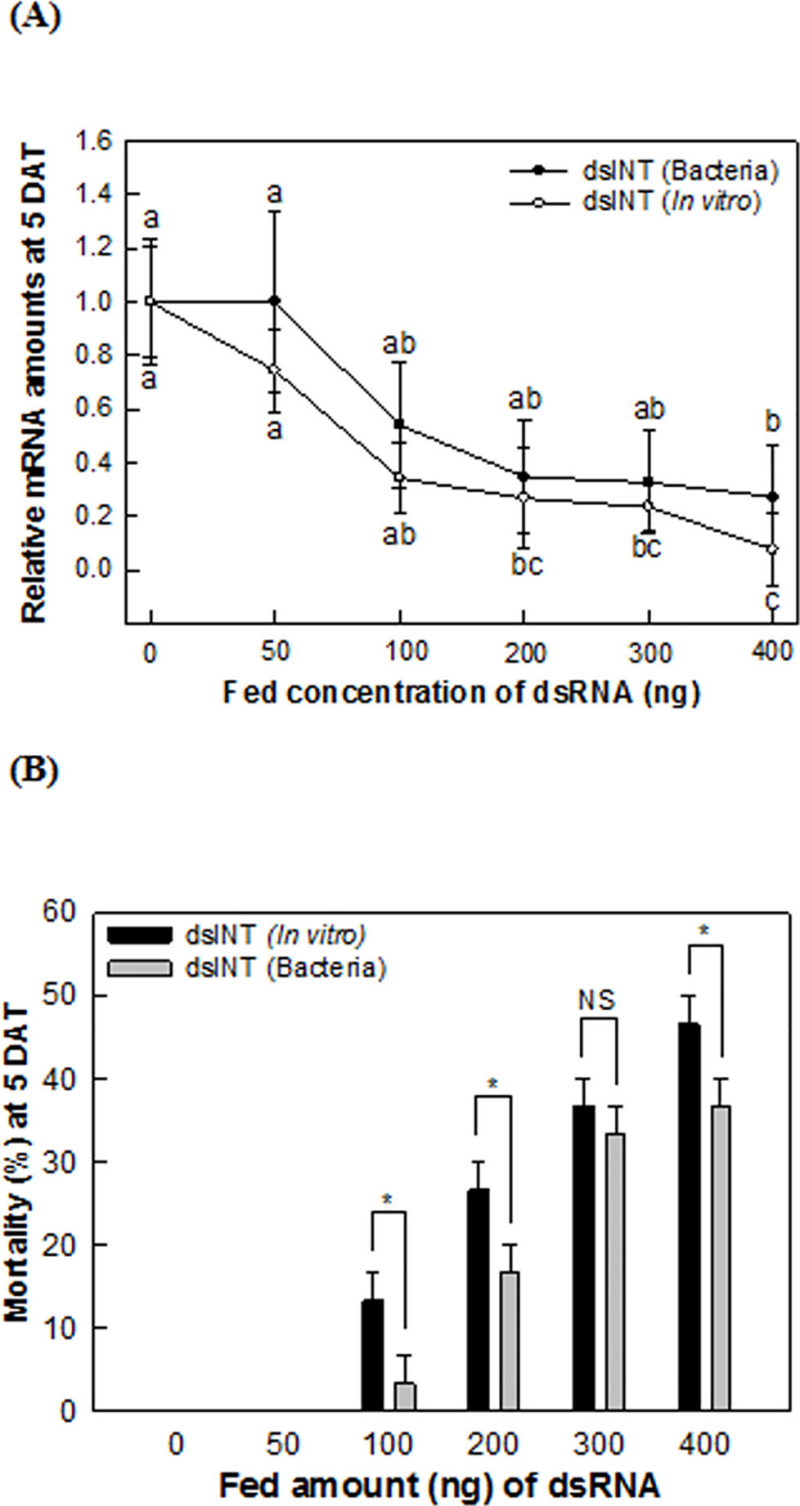
Comparison of two types of dsRNAs prepared by *in vitro* transcription or by the recombinant bacteria in RNA interference efficacy. dsRNA of *in vitro* transcription was orally fed. In dsRNA produced by the bacteria, the recombinant bacteria were orally applied. The number of the bacteria was determined according to the amount of dsRNA produced in the bacteria based on Fig 1B. (A) Suppression of target gene (‘SeINT’) expression levels. At 5 days after treatment (‘DAT’), total RNA was extracted and assessed in the amount of SeINT transcripts. (B) Toxic effects of *in vitro* dsRNA and bacterial dsRNA on young larvae of *S*. *exigua*. Mortality was measured at 5 DAT. Different letters above standard deviation bars indicated significant difference among means at Type Ι error = 0.05 (LSD test). ‘NS’ represents not significant, but ‘*’ indicates significant differences among means at Type I error = 0.05.

The efficacy of dsRNA produced by the recombinant bacteria was compared with that of *in vitro* prepared dsRNA (Fig 3). Both treatments suppressed *SeINT* transcript levels. However, RNAi efficiency appeared to be greater in the treatment using the *in vitro* prepared dsRNA especially at a high dose (400 ng per larva) (Fig 3A). The insecticidal activity was also greater in the treatment using dsRNA prepared by *in vitro* transcription in most dose treatments (Fig 3B).

### Pretreatment effect of the dsRNA-expressing bacteria on the insecticidal activity

**Fig 4:**
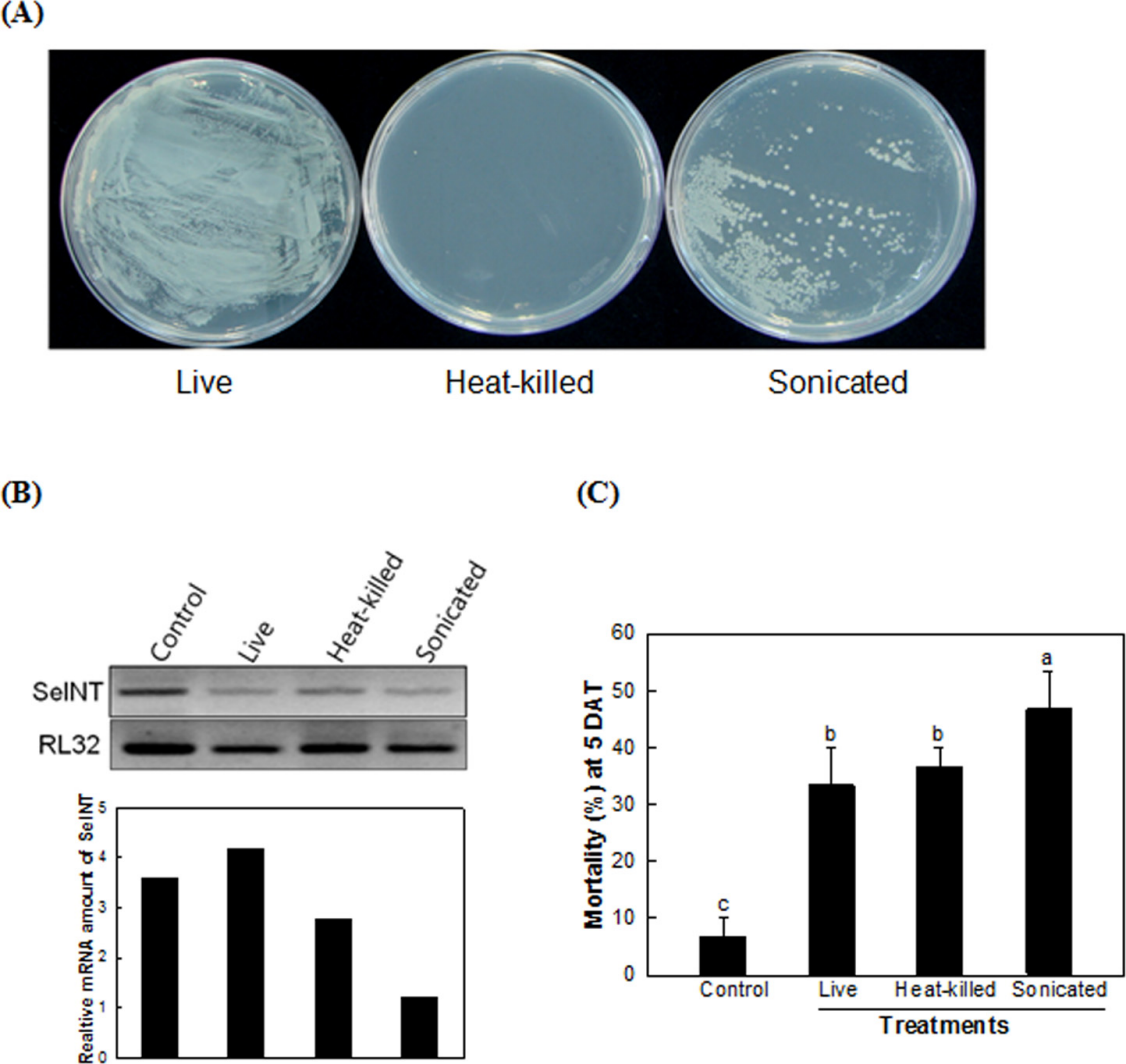
Pretreatment effect of dsINT-expressing *E*. *coli* on its insecticidal activity. (A) Two different pretreatments of recombinant bacteria and their survival: heat-killing (100^°^C for 100 min) and sonication. After pretreatment, the bacterial suspension was plated on LB+AMP plate and cultured for 15 h at 37^°^C. (B) Suppression of SeINT transcript levels with increase of bacterial doses. The expression was assessed by RT-PCR. Expression of a ribosomal gene, RL32, was assessed to confirm the cDNA integrity. (C) Oral toxicity of the recombinant bacteria against young larvae of *S*. *exigua*. Each larva was fed 10^7^ bacteria. Mortality was measured after 5 days of treatment (‘DAT’). Different letters above standard deviation bars indicate significant difference among means at Type Ι error = 0.05 (LSD test).

The lower efficiency of the transformed bacteria expressing dsRNA might be caused by a physical hindrance of the bacterial cell wall, which prevented the release of dsRNA into insect gut lumen. To facilitate the release of dsRNA from the bacteria, the bacteria were treated with heat or sonication (Fig 4A). Heat treatment killed all bacteria, while the sonication gave some significant damage on the bacteria as seen in the reduced number of the bacterial colonies. All transformed bacterial treatments reduced the *SeINT* expression level (Fig 4B). Between these two pretreatments, sonication significantly enhanced the insecticidal activity of the dsINT-expressing *E*. *coli* (Fig 4C). Heat-killed bacteria did not differ with the live bacteria in their insecticidal activities.

### Damage on the midgut epithelium by feeding dsINT-expressing bacteria

**Fig 5:**
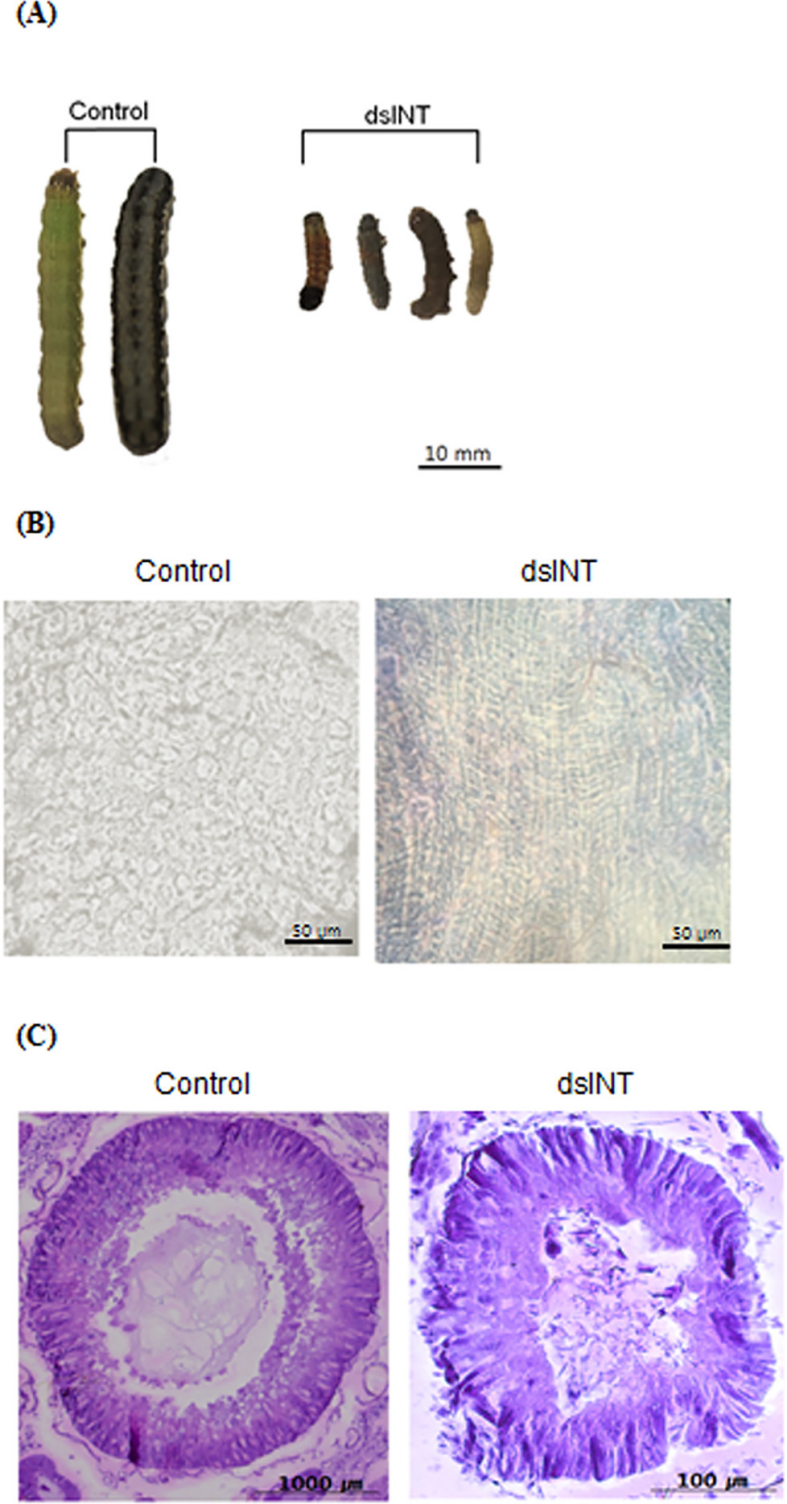
Effect of dsINT-expressing *E*. *coli* on development of *S*. *exigua* and on the midgut epithelium. (A) Effect of the recombinant bacteria on larval development. Control used the bacteria transformed with empty vector. Each larva was fed 10^7^ cells and incubated at 25°C. The pictures were taken at 5 days after treatment. (B) Cytotoxic effect of dsINT expressing bacteria on midgut epithelium. The tissues were stained with 0.1% trypan blue. Stained cells indicate “dead”. (C) Disruption of midgut epithelium in cell-to-cell contacts by oral treatment of dsINT-expressing *E*. *coli*.

The larvae fed with dsINT-expressing bacteria exhibited a delayed development with significantly reduced body size (Fig 5A). This suggested that dsINT might disrupt the integrity of the midgut epithelium to inhibit nutritional digestion and absorption. When the midgut was isolated from the larvae fed with the recombinant bacteria, it contained more number of dead cells (stained by trypan blue in Fig 5B) compared to the midgut epithelium isolated from the control larvae. Cell-to-cell connections in the midgut epithelium were analyzed by microsectioning (Fig 5C). When the guts were cross-sectioned, the midgut size was much different, in which the midgut isolated from the recombinant bacteria-treated larvae appeared to be about 10 times smaller in diameter than the midgut of the control larvae. Furthermore, the midgut epithelium cells were loosely connected in the transformed bacterial treatment, while the midgut cells appeared to be compactly joined in the control.

### Enhancement of Bt toxin efficacy by dsINT-expressing *E*. *coli* against *S*. *exigua*

**Fig 6:**
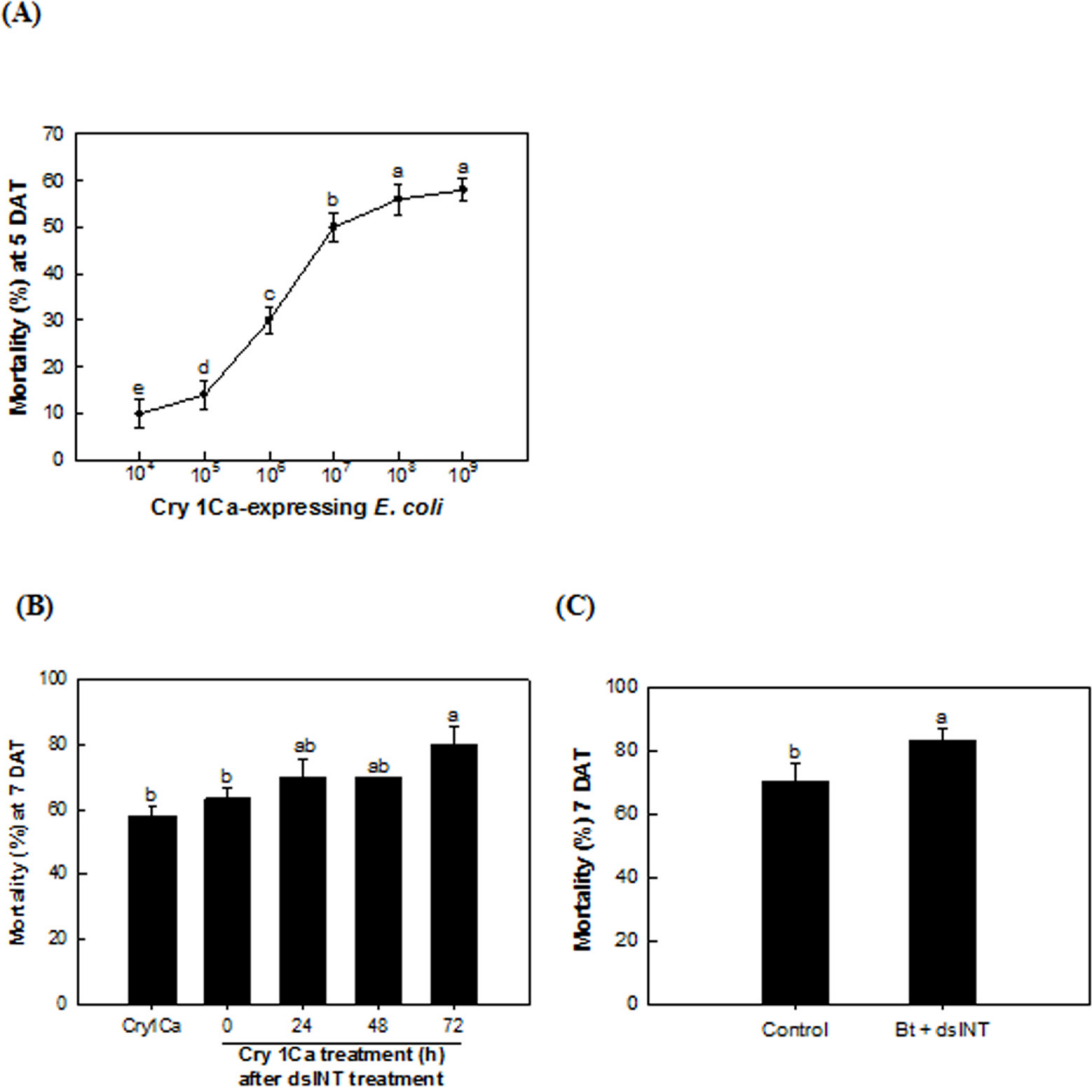
Enhancement of dsINT-expressing *E*. *coli* on Bt Cry toxin toxicity against *S*. *exigua*. (A) Toxicity of Cry1Ca-expressing *E*. *coli* against young larvae. Each treatment used 10 larvae with three replications. Mortality was measured at 5 days after treatment (‘DAT’). (B) Different susceptibilities of dsINT bacteria (10^7^ cells per larva)-treated larvae to Cry1Ca-expressing bacteria according to the pretreatment period. Mortality was measured at 7 days after dsINT-bacterial treatment. (C) Effect of dsINT-expressing *E*. *coli* on toxicity of a commercial Bt (*B*. *thuringiensis* subsp. *aizawai*, Xentari, 1,000 ppm). ‘Bt’ treatment represents no dsINT treatment. ‘Bt+dsINT’ represents a mixture treatment of Bt treatment at 48 h after dsINT bacterial treatment. Mortality was measured at 7 days after dsINT-bacterial treatment. Each treatment used 10 larvae with three replications. Different letters above standard deviation bars indicate significant difference among means at Type Ι error = 0.05 (LSD test).

Despite the sonication pretreatment, the dsINT-expressing *E*. *coli* gave less than 50% mortality against *S*. *exigua* larvae. To increase the larval mortality, the dsRNA treatment was coupled with Bt toxin treatment (Fig 6). Expression of Cry1Ca toxin highly efficient to *S*. *exigua* [28] was induced in a recombinant *E*. *coli* and the toxin protein was confirmed from the protein extract of the recombinant bacteria (S1 Fig). *E*. *coli* expressing Cry1Ca gave a maximal 58% mortality at 10^8^ cells/larva (Fig 6A). When the dsINT-expressing bacteria were treated with the Cry1Ca-expressing bacteria, the insecticidal efficacy of the Bt toxin was significantly enhanced up to about 80% (Fig 6B). The enhanced efficacy of the Cry1Ca-expressing bacteria was dependent on the preincubation time after the dsINT-expressing bacterial treatment. The significant enhanced efficacy was observed at 72 h preincubation after the dsINT-expressing bacterial treatment compared to control treatment. However, the enhanced efficacy was not significantly different among pretreatment periods (24–72 h). The enhanced toxicity by the dsINT-expressing bacteria was also observed with a commercial Bt formulation treatment (Fig 6C).

## Discussion

This study showed an insecticidal activity of a transformed *E*. *coli* expressing dsRNA specific to an integrin β subunit of *S*. *exigua*. The transformed *E*. *coli* expressing dsINT gave significant oral insecticidal activity against young larval instars and delayed the larval development. Some of the larvae treated with the bacteria exhibited a miniature body form probably due to malnutrition induced by the midgut damage caused by dsINT. The treated larvae had significantly reduced level of *SeINT* expression by the specific RNAi. This physiological alteration was reported in an earlier study using feeding dsINT prepared by *in vitro* transcription reaction [27]. To control insect pest using dsRNA, *E*. *coli* has been used to synthesize dsRNA and to formulate dsRNA product to be released in the gut after feeding by digestive enzyme(s) or disturbing factor(s) of bacterial cell wall and membrane. Bacteria-induced RNAi was first demonstrated in a genetic interference experiment performed in *Caenorhabditis elegans* [29]. To overcome some inefficiency, a bacterium engineered in deleting RNase ΙΙΙ was constructed and improved the RNAi efficiency [30]. Application of this technology to control insect pests has been tried against a coleopteran insect, *L*. *decemlineata*, and showed effective suppressions of five different target genes, which led to significant mortality and developmental retardation [10]. This technology was also applied to *S*. *exigua* by targeting a chitin synthase A (CHSA) gene. Tian et al. [25] showed that *S*. *exigua* fed with *E*. *coli* expressing dsRNA specific to CHSA gene resulted in significant mortalities at 62–88%, in which younger developmental stages were more susceptible to the RNAi treatment than old instars. Similarly, our current study showed that *S*. *exigua* fed with *E*. *coli* expressing dsINT suffered significant mortality, in which young larvae were more susceptible than later instars. However, the control efficacy to kill *S*. *exigua* was less than 50%. The lower control efficacy compared to the similar study may be explained by the different target genes in these two studies. Two different chitin synthase genes (CHSA and CHSB) have been reported, in which CHSA is expressed in all ectodermal origin tissues, which CHSB is expressed in midgut tissue [31]. Considering massive requirement of CHSA during insect development, the suppression of CHSA by RNAi can be understood in its potency to give significant lethality to *S*. *exigua*. Thus, the selection of target gene for development of dsRNA insecticides would be a crucial factor to improve the control efficacy. However, the combinational action of dsRNA with Bt toxicity may be varied with different target genes.

The recombinant bacterial treatment was less effective to kill target insects compared to the treatment using *in vitro* synthesized dsRNA. The transformed bacteria expressed the dsRNA and the produced dsRNA amount was proportional to the bacterial cell number. Based on the total dsRNA amount and the bacterial cell counts, our estimate showed that one recombinant *E*. *coli* produced 2.8 ± 0.1 pg of dsRNA. This indicates 350 ng of dsRNA to be effective to give a maximal insecticidal activity by feeding the transformed *E*. *coli* because the maximal mortality was obtained from the bacterial treatment at 10^7^ cells per larva. If all dsRNA molecules were released in the gut after feeding 10^7^ bacterial cells, 8.68 x 10^11^ molecules of dsRNA (based on 249,191.9 molecular weight of dsINT) would be estimated to enter target cells. However, these dsRNA molecules may be faced to an attack by dsRNA-degrading enzymes. Degradation of dsRNA in the gut has been reported in several insects including *Bombyx mori* [32] and *Lygus lineolaris* [33]. In addition, salivary secretion during feeding may secrete the dsRNA-degrading factor as seen in a sucking insect, *Acyrthosiphon pisum* [34]. Thus, the actual number of dsRNA molecules released from the transformed *E*. *coli* may be remarkably reduced by degrading factor in the gut of *S*. *exigua*. Furthermore, the release of dsRNA synthesized in the recombinant bacteria may be a limiting factor to give an ideal insecticidal activity. In an effort to increase insecticidal activity of the bacteria expressing dsINT in our current study, we tried to improve the efficiency of dsRNA release from the live *E*. *coli* by pre-treating the bacteria with heat or ultra-sonication to disrupt the bacterial cell wall and membrane. While there was no difference in insecticidal activity between live and heat-killed bacteria, a pretreatment of sonication gave significantly enhanced efficacy to kill insects. Killing bacteria expressing dsRNA was tested in a shrimp for an aquatic environmental safety purpose, in which the pretreated bacteria effectively suppressed target gene expression [35]. However, little attention has been gained in the release route of dsRNA from the transformed bacteria. Our current data suggest that disruption of bacterial cell wall and membrane integrity by sonication pretreatment helps the release of dsRNA from the bacteria, which would be required for an optimal application of dsRNA-expressing bacteria to control insect pests. Then the free dsRNAs could enter the midgut epithelial cells of S. exigua. In lepidopteran insects, RNAi efficiency varies with species, tissues, and target genes [36]. *S*. *exigua* is one of the most tested insects for RNAi using dsRNA in both systematic and environmental RNAis [20,25,27,37]. Especially, a homologue of the *Caenorhabditis elegans* systemic RNA interference deficient-1 (*Sid-1*) gene, which is responsible for the systemic spread of dsRNA in the worm, has been identified in *S*. *exigua* [25].

Insecticidal activity induced by RNAi of *SeINT* following the transformed bacterial treatment may be primarily caused by a serious impairment of the midgut epithelium. Our previous study [27] showed that RNAi of *SeINT* led to a significant immune suppression and developmental retardation. Integrin is a transmembrane protein and plays a crucial role in cell-cell or cell-ECM contacts for immunity, growth, and communication [26]. It forms a heterodimer with functional combinations of α and β subunits. A single gene RNAi of integrin subunits in our current assay may fail to form functional joining of integrin subunits in the midgut epithelium, which is essential for insect digestion and absorption of nutrients [38]. The retarded development and larval mortality by feeding the *E*. *coli* expressing dsINT may be understood by the damage of the digestive system.

Combinational effect of the bacteria expressing *dsINT* on the efficacy of Bt toxin was demonstrated in this study. Bt Cry toxins induce the fatal cell death of the midgut epithelium, which leads to septicemia [39,40]. A specific cadherin receptor is associated with Bt toxicity in *S*. *exigua*, in which Cry1Ca is highly toxic than other Cry toxins [28]. To give a significant combinational activity of the bacteria expressing dsINT to Bt toxin, the target gene (*SeINT*) suppression should be preceded before the Bt treatment because the Bt toxicity increased with the elapse time after feeding the dsINT-expressing bacteria. A similar bacterial treatment study in *S*. *exigua* showed that it took more than 7 days after the feeding [25]. Our current study showed that the effective RNAi effect was detected at 3 days after the bacterial feeding treatment to *S*. *exigua* larvae, at which the Bt efficacy was significantly enhanced. This provides a novel technique to enhance insecticidal efficacy of current Bt crops by supplementation with expression of dsRNA specific to integrin.

These results show that the transformed *E*. *coli* expressing dsINT has a significant insecticidal activity by oral application. This study also indicated that the bacteria expressing dsINT potentiate Bt toxicity. This may be also applicable to control *S*. *exigua* populations, which become resistant to Bt.

## Materials and Methods

### Insect rearing

Larvae of *S*. *exigua* were reared on an artificial diet [41] at 25°C, 16:8 h (L:D) photoperiod and 60 ± 5% relative humidity. Adults were supplied with 10% sucrose solution. However, insecticidal bioassays used Chinese cabbage for diet to deliver dsRNA in an oral route.

#### RNA extraction and RT-PCR

Total RNA was extracted using Trizol reagent (Invitrogen, Carlsbad, CA, USA) according to manufacturer’s instruction. An extraction used 20 young larvae (1st-3rd instar), three 4th instar larvae or one 5th instar larva. The extracted RNA was treated with a RNase-free DNase (Bioneer, Seoul, Korea) to degrade any genomic DNA contamination. RNA extract (1 μg per reaction) was incubated at 70°C for 3 min and then used for synthesis of cDNA using RT-premix (Intron Biotechnology, Seoul, Korea). The synthesized cDNA was used for PCR amplification with SeITG-specific forward primer (5’-TCTAGACAGCTTGCCAGTGTTTGAAG-3’) and reverse primer (5’-AAGCTTCCGTTCCTTCTGTGCTCTAAT-3’). PCR was performed with 35 cycles after an initial denaturation at 94^°^C for 5 min. Each cycle consisted of denaturation at 94^°^C for 30 s, annealing at 50^°^C for 30 s and extension at 72^°^C for 1 min. The PCR reaction was ended with an extension step at 72^°^C for 10 min. Quantitative PCR (qPCR) used SYBR Green Realtime PCR master mixture (Toyobo, Osaka, Japan) by 7500 real time PCR system according to the manufacture’s instruction. The reaction mixture (20 μL) included 5 pmol of primers used in RT-PCR as described above, and 50 ng of template cDNA. After activation of Hot-start Taq DNA polymerase at 94^°^C for 15 min, the reaction was amplified with 35 cycles of 30 sec at 94^°^C, 30 sec at 50^°^C, and 1 min at 72^°^C with a final extension for 5 min at 72^°^C. Fluorescence values were measured and amplification plots were generated in real time by an Exicycler TM program. Quantitative analysis of amplification was done using the comparative C_T_ method [42].

#### Cloning of dsRNA specific to *SeINT*

To construct a recombinant plasmid to express dsRNA corresponding to a fragment (410 bp, Fig 1A) at the extracellular domain of integrin beta subunit, the amplified DNA (above) was digested with *Xba*Ι and *Hin*dΙΙΙ, and inserted into L4440 vector kindly donated from Seung Jae Lee (Pohang University of Science and Technology, Pohang, Korea). The L4440 plasmid has two T7 promoters in an inverted orientation flanking the multiple cloning sites. The recombinant vector L4440-SeINT was transformed into a competent cell of *Escherichia coli* HT115 (donated from SJL) lacking RNase ΙΙΙ. This bacterium was designed to be induced to express T7 RNA polymerase in the presence of IPTG.

#### Preparation of transformed bacteria and over-expression of dsRNA

To produce dsRNA, the bacteria transformed with L4440-SeINT were grown in Luria-Bertani (LB) containing 100 μg/mL ampicillin (LB-AMP) at 37^°^C for 16 h with 250 rpm shaking rate. The cultured broth (5 mL) was added to 500 mL of LB medium and allowed to grow to OD_600_ = 0.6~0.7. Expression of T7 RNA polymerase gene was induced by an addition of IPTG to 0.1 mM and the bacteria were incubated with shaking for 4 h at 37°C. The expressed dsRNA was extracted using a RNA extraction mini kit (Qiagen Korea, Seoul, Korea). The purity of the synthesized dsRNA was confirmed by electrophoresis on 1% agarose gel. To assess insecticidal activity of the recombinant bacteria expressing dsRNA, IPTG-induced cultures were pelleted by centrifugation and resuspended in the same culture medium, and applied to cabbage leaves for feeding treatment (see below).

#### Quantification of dsRNA produced by the transformed *E*. *coli*

Total RNA from *E*. *coli* cells was isolated as described above. To quantify the extracted dsRNA, the dsRNA sample was separated on 1% agarose gel and visualized with ethidium bromide. A standard curve was generated with the known amounts of purified dsRNA synthesized by *in vitro* transcription method [43] by comparing them with the gel band intensities estimated by an image analyzer (Bio-Rad Korea, Seoul, Korea). The RNA band intensity was read in pixels by Image Lab software (Bio-Rad Korea).

#### Pretreatments of the transformed bacteria

The bacteria expressing dsRNA specific to *SeINT* were cultured at 37°C and over-expressed as described above. Bacterial cells were harvested by centrifugation at 2,720 x g for 30 min at 4°C and resuspended in the fresh culture medium. Two different pretreatments were applied to the bacterial cells. A heat treatment used 100°C for 10 min. A sonication treatment used an ultrasonicator (Bandelin Sonoplus, Berlin, Germany) at 95% intensity with 10 cycle of 10 min burst separated by 2 min gaps. The bacterial viability was assessed by plating 100 μL of the treated bacterial sample on LB+AMP.

#### Bioassay of the transformed bacteria expressing dsRNA

To assess the insecticidal activity of the bacteria expressing dsRNA specific to *SeINT*, different instar larvae of *S*. *exigua* were used in the feeding bioassay. The cabbage was cut into two sizes: the smaller one with 20 mm diameter was used for assessment of 1st and 2nd instar larvae, while the larger one with 30 mm diameter was used for the older instar larvae. Each leaf disc was overlaid with 150 μL suspension of treatment (recombinant vector-containing) or control (non-recombinant vector-containing) bacterial suspension. Each treatment used 10 larvae with three replications. Fresh untreated cabbage was supplied after 24 h feeding of the treated leaf disc. For a long-term exposure experiment, the treated diet was replaced every 24 h. Insect mortality was measured daily for 7 days after treatment. Assessment of RNAi efficiency used qPCR as described above.

#### Examination of the midgut epithelium

To assess any damage on larval midgut epithelium, each 3rd instar larva was fed with the bacteria (10^7^ cells per larva) expressing dsRNA specific to SeINT. Control larvae were fed with the bacteria transformed with a non-recombinant vector. At 7 days after the transformed bacterial treatment, the larvae were dissected to collect the midgut. After cutting the midgut longitudinally, the midgut content was thoroughly washed with 100 mM phosphate buffer saline (PBS, pH 7.4). The washed rectangular midgut was soaked into 0.1% trypan blue (Sigma-Aldrich Korea, Seoul, Korea) solution for 10 min and destained three times in PBS for each 10 min. The resulting tissues were observed under a phase contrast microscope (BX41, Olympus, Tokyo, Japan) at 200 x magnification. To assess the ultrastructure of the midgut epithelium, the prepared midgut tissue was fixed for 16 h with Bouin fixative (7.5 mL saturated picric acid, 2.5 mL 40% formalin, 0.5 mL acetic acid glacial) and dehydrated by an ascending ethanol series from 30 to 100%. Then, the tissues were cleared with xylene to be infiltrated with paraffin (58 ± 2°C melting point, Merck Korea, Seoul, Korea). The tissue-embedded paraffin was sectioned with 5 μm width using Buffalo microtome (Nippon Optical Works, Tokyo, Japan). The paraffin section was attached on the slide glass covered with 10% albumin. After deparaffin processes using a descending ethanol series, the tissues were stained with hematoxylin (1 g hematoxylin, 1.5 g ammonium alum, 50 mL ethanol in 100 mL of 50% glycerol) and eosin Y solution (0.1 g eosin and 0.5 mL acetic acid in 100 mL of 70% ethanol). These samples were pictured with a phase contrast microscope (BX41, Olympus) equipped with a color digital camera INFINITY1 (AZ microscope, Cheapside, London, UK).

#### Preparation of *E*. *coli* expressing Bt Cry1Ca

A Bt clone containing Cry toxin (Cry1Ca) was kindly donated from Yeon Ho Jae (Seoul National University, Seoul, Korea). A partial N-terminal Cry toxin (935 residues) was cloned at *Sma*I and *Not*I sites of pGEX-4T-1 expression vector (GSL Biotech, Chicago, IL, USA) and expressed under IPTG induction as described above. For a dose-mortality assessment, different cell numbers of the bacteria were prepared and applied on cabbage leaf discs and fed to the 3rd instar larvae. Subsequently, the determined minimal dose to give a maximal mortality was used to assess a combinational effect of the *E*. *coli* bacteria expressing dsRNA specific to *SeINT* on the Bt toxicity. To determine any sensitive stage to Bt toxin, the Cry1Ca-expressing bacteria were treated at different time points after feeding *E*. *coli* expressing dsRNA. Each treatment used 10 larvae with three replications. Mortality was measured at 7 days after the dsRNA-expressing bacterial treatment.

#### Statistical analysis

All bioassays were performed in three independent biological replicates and plotted by mean ± standard deviation using Sigma plot. Means were compared by least squared difference (LSD) test of ANOVA using PROC GLM of SAS program [44] at Type I error = 0.05.

## Supporting Information

S1 Fig Production of Cry toxin protein of a recombinant E. coli transformed with pGEX-4T-1 vector recombined with a partial Cry1Ca toxin gene (N-terminal 935 residues). A fusion protein (ca. 120 kDa) with GST was detected with a Western analysis using Cry1Ca antibody. A faint band at 65 kDa may be a degraded toxin.
(TIF)
