# Supplementary figures and images for: A Transformed Bacterium Expressing Double-Stranded RNA Specific to Integrin β1 Enhances Bt Toxin Efficacy against a Polyphagous Insect Pest, Spodoptera exigua
Source: PLoS One. 2015 Jul 14;10(7):e0132631. doi: 10.1371/journal.pone.0132631 (PMC4501564; doi:10.1371/journal.pone.0132631)

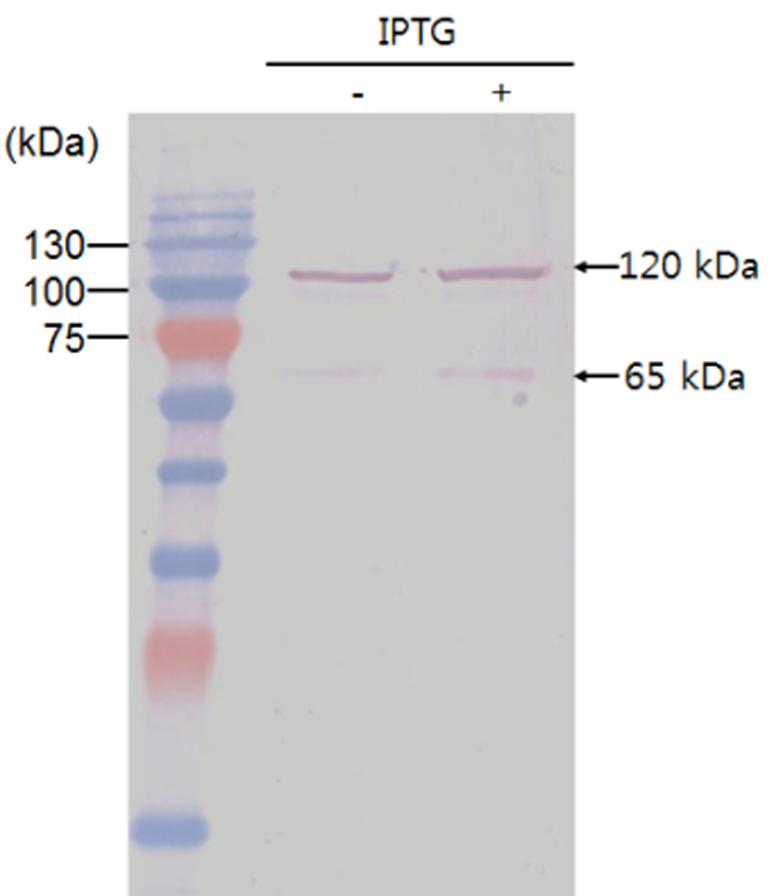

Supplement: S1 Fig — A fusion protein (ca. 120 kDa) with GST was detected with a Western analysis using Cry1Ca antibody. A faint band at 65 kDa may be a degraded toxin. (TIF) [file pone.0132631.s001.tif]
